# Corrosion Stiction in Automotive Braking Systems

**DOI:** 10.3390/ma16103710

**Published:** 2023-05-13

**Authors:** Michele Motta, Lorenzo Fedrizzi, Francesco Andreatta

**Affiliations:** Polytechnic Department of Engineering and Architecture, University of Udine, Via del Cotonificio 108, 33100 Udine, Italy; motta.michele@spes.uniud.it (M.M.); lorenzo.fedrizzi@uniud.it (L.F.)

**Keywords:** braking system, friction material, corrosion, stiction

## Abstract

This review paper targets the corrosion-stiction phenomenon that can occur in automotive braking systems under static conditions in aggressive environments. The corrosion of gray cast iron discs can lead to a strong adhesion of the brake pad at the pad/disc interface that can impair the reliability and performance of the braking system. The main constituents of friction materials are initially reviewed in order to highlight the complexity of a brake pad. Corrosion-related phenomena, including stiction and stick-slip, are considered in detail to discuss the complex effect of the chemical and physical properties of friction materials on these phenomena. In addition, testing methods to evaluate the susceptibility to corrosion stiction are reviewed in this work. Electrochemical methods, including potentiodynamic polarization and electrochemical impedance spectroscopy, are useful tools for a better understanding of corrosion stiction. The development of friction materials with low susceptibility to stiction should follow a complementary approach targeting an accurate selection of the constituents, control of local conditions at the pad–disc interface, and the use of specific additives or surface treatments to reduce the corrosion susceptibility of gray cast-iron rotors.

## 1. Introduction

The rotor–brake pad assembly for application in automotive braking systems is a complex multimaterial system subjected to several aggressive environments, including rain, snow, and chlorides during winter driving [1]. As a result, corrosion issues involving the rotor–brake pad assembly, in particular for cast-iron rotors, can negatively affect the performance, safety, and reliability of the braking system. Under highly oxidative conditions, electrochemical processes occurring at the gray cast-iron rotor surface can generate corrosion products (iron oxides) that can penetrate the brake pad material through the intrinsic porosity of the friction material employed for the production of the brake pad. Under static conditions, for instance, when the braking pad is activated, this can lead to stiction which consists of a strong adhesion of the pad material on the rotor [2,3]. This phenomenon can lead to damage to the brake-pad material after releasing the parking brake. In some cases, stiction phenomena can severely affect the performance of the braking system leading, in the worst cases, to the impossibility of driving the vehicle. Another corrosion-related issue in automotive braking systems is the stick-slip phenomenon that can generate noise and vibrations in the braking system [4].

The automotive industry is strongly oriented towards the replacement of combustion-engine vehicles with electric vehicles. In electric (BEV), hybrid (HEV), and plug-in hybrid (PHEV) vehicles, braking is demanded by the regenerative braking system which is used to recover kinetic energy during braking. Therefore, the rotor–brake pad assembly is strongly undersized and inherently more susceptible to corrosion-related problems than in conventional combustion-engine vehicles. After a brief discussion about the main constituents of friction material, this paper reviews the main literature concerning corrosion phenomena in automotive braking systems focusing mainly on the stiction phenomenon and its control. In addition, testing methods to evaluate the susceptibility to stiction are considered both at the industrial level and laboratory level in order to highlight the main parameters controlling stiction (environmental conditions and properties of the rotor and friction material).

## 2. Brake Pad Constituents

**Figure 1:**
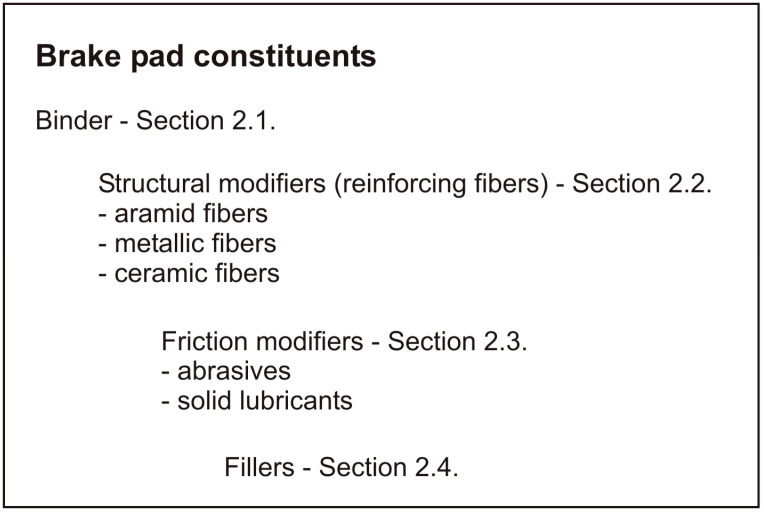
Brake pad constituents.

Friction materials employed in automotive braking systems are usually composed of more than 20 components. Several hundred ingredients have been reported in the literature for being commonly used to tailor the friction-material properties [1]. Nevertheless, brake pad constituents can be classified into four main categories (Figure 1), namely:

1. Binder: to hold the different components together;
2. Structural modifiers: typically in the form of reinforcing fibers to provide mechanical strength;
3. Friction modifiers: a combination of lubricants and abrasives to adjust the friction properties of the brake pad;
4. Fillers: to facilitate the production of the brake pads and reduce their cost.

The different constituents of a friction material can provide different functions including [5]:

- to maintain a high friction coefficient with the rotor during braking;
- to retain sufficient stability at high temperatures;
- to maintain a stable friction coefficient under different braking conditions.

The coefficient of static friction is defined as the ratio between the tangential force to start the relative motion between two bodies and the normal force keeping them in contact [6]. When the bodies are in relative motion with respect to each other, this ratio is referred to as the coefficient of dynamic friction. The friction coefficient is a function of time (or sliding distance) [6,7]. It typically displays an initial transition regime with linear behavior until a peak is reached [7]. This transition regime is followed by a steady state regime corresponding to the stabilization of the friction coefficient [7]. The peak at the end of the transition regime corresponds to the coefficient of static friction, while the steady-state regime is associated with the coefficient of dynamic friction [7].

The synergistic effect existing between the different ingredients and their influence on the friction proprieties is extremely complex [8]. Herbert Frood is credited with the invention of the first brake-lining materials in 1897 [5,9]. In 1908, he also introduced, for the first time, the use of a friction material made by the combination of asbestos, brass wire, and resin [1]. Asbestos was a cheap material, thermally stable up to 500 °C with heat resistance, mechanical strength, and processability [9]. Since asbestos is a carcinogenic compound, its elimination led to a sequence of evolutionary stages in the formulation of friction materials [10]. This was mainly accomplished with the introduction of different types of reinforcing fibers [8]. Although this review is focusing on corrosion stiction, the main constituents of friction materials are briefly considered because they can strongly affect the stiction behavior at the pad–disc interface.

### 2.1. Binder

The function of a binder is to ensure the integrity of the friction material under mechanical and thermal stresses and to hold together the different components of the brake pad composite material [1]. At high temperatures, binder deformation and decomposition can lead to modification of the shear-stress distribution at the braking interface. Low frictional output and fracture of the sample may be the results of thermal overloading at the friction material surface. Furthermore, a binder with poor thermal stability will present a low frictional output due to the deformation and decomposition of the composite material surface.

Resin-based materials are the most employed binders in friction materials for commercial vehicles. Flexural strength, tensile strength, hardness, and storage modulus are typically driving the selection of the binder. Phenolic resins are the most common binders for the friction-material industry for their favorable performance/price ratio and good wetting capability with most of the friction-material ingredients [11]. Nevertheless, phenolic resins are brittle and display low impact resistance, have low thermal resistance at elevated temperatures, and short shelf life [11]. Phenolic resins are thermoset polymers that can be classified according to the phenol/formaldehyde ratio and the reaction conditions (acid or alkaline). Novolac resins, typically employed in friction materials, are obtained using an acid catalyst in the presence of a high phenol/formaldehyde ratio. A commercial phenolic resin shows a glass transition temperature in the range of 200–300 °C with carbonization initiating at 450 °C. This leads to a decrease in the density of the friction material associated with an increase in its porosity and loss of structural integrity. Degradation of the phenolic resin may also result in a decrease in the coefficient of friction [12]. Therefore, different modified versions of phenolic resins are employed for the production of friction materials. In a recent work by Cai et al. [11], phenolic resin composites were modified with nitrile rubber and boron to prepare different friction materials. A friction material with 17.7% by volume boron showed a high cross-linking degree with improved thermal resistance, reduced brittleness, and low flexural strength. In addition, flexural strength and porosity were inversely proportional [11].

The porosity of the composite can be easily tailored by varying its binder content. Friction materials with high porosity tend to decrease noise and improve heat dissipation during braking. This can also affect the wear rate, which is the result of a complex combination between the thermal stability of the resin and its mechanical properties [11,13]. As an example, Kim et al. [14] showed that the use of an aralkylether-modified resin improved heat resistance but was accompanied by an increase in the wear rate of the friction material. In addition, Shin et al. [15] reported that polyimide resins, which can be employed to improve fade resistance, displayed high wear rates due to low stiffness at high temperatures. Furthermore, a porous structure may allow the lubricant to easily reach and flow in the contact zone with the brake disk, thus, reducing the temperature and wear rate of the composite in the heat-effected zone [16].

In addition to phenolic resins, other types of resin can be also used as friction-material binders [1]. These include condensed polynuclear aromatic resin, silicon-modified resin, cyanate resin, epoxy-modified resin, and thermoplastic polyimide resin [1]. Gurunath et al. [17] showed that the ring-opening polymerization of polybenzoxazines can be employed for the formation of friction materials with unlimited shelf life, low moisture absorption, and minimal volumetric change, with low or no formation of byproducts in the curing reaction. Carlevaris et al. [18] showed that bisphenol A and bisphenol F benzoxazine-based resins can significantly reduce particulate emissions as compared to traditional binders while preserving their tribological properties.

### 2.2. Reinforcing Fibers

In general, a total of ingredients ranging from 5 to 25% is commonly used in the form of fibers to increase the mechanical resistance, hardness, and resilience of the composite friction material. The main types of reinforcements are aramid, metallic, and glass or ceramic fibers.

#### 2.2.1. Aramid Fibers

Aramid fibers are obtained from the condensation of terephthalic or isophthalic acids and m- or p-phenylenediamine. These types of fibers present high thermal stability with a decomposition temperature of 427 °C in air, low weight, excellent stiffness-to-weight ratio, and superior antifade, wear, and friction stabilization properties [1,14,19]. A drawback of aramid fibers is that they are relatively soft and are not effective for supporting the braking load [8]. Aramid fibers can be used as powder, but they are usually preferred as pulp with a fibrillary structure. In the form of pulp, they are capable of maintaining the uniformity of the composite-material mixture during the pad-molding process [14]. Moreover, they tend to form a molten viscous glassy film during sliding, promoting the adhesion of the pad to the rotor and improving fade resistance [20].

#### 2.2.2. Metallic Fibers

Different metallic constituents, including steel, bronze, copper, brass, aluminum, or zinc, may be incorporated in the friction material formulation in the form of fibers, chips, or granules [1,8,21]. Metallic fibers are used to improve the mechanical strength, wear resistance, and thermal diffusivity of the brake pad. Semimetallic friction materials contain metallic fibers (steel wool and copper chips) to combine high mechanical resistance and coefficient of friction with high thermal conductivity to efficiently remove heat from the friction surfaces during braking [22]. Copper is also used in powder form to control heat transport during braking and as a solid lubricant to reduce the fluctuations in the coefficient of friction [9]. This effect is commonly attributed to the high metal conductivity of copper, to the formation of copper oxides at the friction interface during high-temperature sliding, and to the uniform transfer films formed on the disk surface [23].

Although the use of metallic fibers is a common practice, their introduction to the friction material can also present some drawbacks. Since the coefficient of friction is related to the Mohs hardness of the friction material, metallic constituents with high hardness and high metallic adhesion with the brake disc can cause excessive wear of the brake rotor and lead to vibrations and noise during braking. Moreover, the wear rate is strongly related to the compatibility between the metallic fibers in the friction material and the rotor. This was investigated by Jang et al. [21] for different metallic fibers in the friction-material formulation employing a gray cast iron and an A356 aluminum alloy containing 30% SiC by volume. It was found that steel and copper fibers in the friction material tend to decrease the coefficient of friction when the rotor speed is increased. This behavior could result in a sudden increase of the friction force during the final part of the braking, also referred to as antifade, which is often associated with noise and brake judder [21]. Moreover, it was found that the coefficient of friction increased with the applied braking pressure for tests performed with a gray cast-iron rotor, while this was not observed for the aluminum-metal composite rotor [21].

#### 2.2.3. Ceramic Fibers

Ceramic reinforcements such as glass and potassium titanate fibers present high thermal resistance and stability, high strength-weight ratio, and high hardness [1]. Brake pads with a high content of ceramic fibers are also characterized by reduced vibration, dust, and wear compared to other friction materials [1]. Ceramic fibers can have an abrasive effect on the rotor due to their high hardness [1]. However, the work of Han et al. showed that the use of ceramic reinforcements could reduce the wear of the brake rotor and improve fade resistance [24]. This was associated with a lubricating effect generated by the incorporation of the ceramic fibers in the friction layer [24]. Nevertheless, other works show that the increase in the coefficient of friction due to ceramic fibers might also lead to an increase in the wear rate of the friction material [25].

Glass fibers are the most used ceramic reinforcements in nonasbestos organic friction materials due to their high melting point (1430 °C) and favorable combination of thermal and impact resistance [19]. Glass fibers tend to soften above 600 °C and show a strong bonding with the phenolic resin used as a binder [9]. Nevertheless, glass fibers are intrinsically brittle, and often they should be employed in combination with more ductile reinforcements such as aramid fibers. Moreover, they present low thermal conductivity (0.04 W/m K) that can negatively affect the fade performance of the braking system due to limited heat dissipation during braking [26]. A temperature increase during braking may also lead to the thermal decomposition of the binder with an effect on the fade and wear characteristics of the friction material [19]. The type of glass fiber introduced in the friction material can affect its friction behavior [26,27]. Milled glass fibers promote the formation of more compact wear debris around the reinforcement resulting in a higher coefficient of friction as compared to chopped fibers [27]. In addition, it is also shown that the use of dispersed milled glass fibers can result in stiff contact surfaces with friction instability, noisy vibrations, and stick-slip propensity [27].

Potassium titanate reinforcements in the form of whiskers, platelets, or splinters are often used in the formulation of nonasbestos organic friction materials. In particular, crystalline whiskers of potassium titanate with high aspect ratios exhibit an outstanding thermo-oxidative stability (up to about 500 °C), low thermal conductivity, high tensile strength, excellent reinforcing ability, and good friction-wear behavior at a high temperature (200 °C) [1]. The tribolayer formed by friction materials containing potassium titanate fibers exhibits a stable coefficient of friction over time [28,29]. Due to the abrasive effect of the potassium titanate fibers, they are often combined with aramid fibers, improving the wear behavior of the friction material and increasing the stability of the friction film [20,30].

Since reinforcements in the form of whiskers can present health hazards, their replacement with potassium titanate platelets and splinters has been recently addressed [31]. However, it was shown that the thermal stability of the platelets is lower compared to whiskers, and poor adhesion to the binder is reported above its glass transition temperature for platelet-like reinforcements. The use of potassium titanate splinters leads to more uniform transfer films with increased wear resistance attributed to an offsetting effect of the thermal degradation of the binder [31].

### 2.3. Friction Additives

Friction additives instead are added in the formulation of brake-pad materials to control their friction performance [1]. They are usually classified as abrasives and lubricants. The abrasives increase the friction coefficient and the wear rate, while the lubricants display the opposite effect [1]. Lubricants and friction materials are always present in the formulation of a friction material to obtain a stable friction coefficient [1,5,8,32]. Nevertheless, it is important to highlight that a correct mixing of lubricants and abrasives must take into account the environmental issues related to air emissions from friction materials [33,34]. In this perspective, the selection of abrasives, such as alumina (Al_2_O_3_) or silicon carbide (SiC), and lubricants such as graphite or coke, plays a crucial role in finding a compromise between the performance of a braking system and its environmental compatibility [35,36,37].

### 2.4. Fillers

Fillers are commonly employed to improve the manufacturing process of friction materials to modify their performance and reduce their cost [1]. Inorganic fillers, including barium sulfate, calcium carbonate, and mica, are typically present in brake pads. Barium sulfate (barite) improves the heat stability and friction characteristics due to its high melting point (1350 °C) [38,39]. Calcium carbonate may be also used as a cheaper alternative to barite to improve the thermal and fade properties of the friction materials [39]. Vermiculite is another inorganic filler commonly employed in friction material composition [40,41]. Due to its stacked-layer morphology, exfoliated vermiculite is used to enhance stability, durability, and high-temperature resistance of the friction material and to reduce brake noise [1,41]. Satapathy et al. [40] reported that friction materials containing vermiculite display a decrease in hardness and good performance in terms of thermal resistance and friction behavior. Molybdenum trioxide is another inorganic additive that can be employed to reduce fading and cracking of the friction material under high thermal stress [1]. Organic fillers such as rubbers and cashew dust, also known as friction dust, are also used to reduce brake noise and fluctuations of the friction coefficient at high temperatures [42]. Friction dust is obtained from cashew nut-shell liquid, which is mainly composed of anacardic acid [42]. Furthermore, friction dust contains unsaturated phenolic groups, which render this additive strongly compatible with the phenolic binders typically employed in friction materials [8].

## 3. Environmental Impact of Corrosion on Braking Systems

The particulate matter produced by vehicle brake pads during braking is recognized as a potentially critical issue linked to the release into the environment of particles not deriving from combustion processes (nonexhaust particle emissions) in the transport sector [43,44,45]. In particular, the release of PM10 due to wear of the friction material of automotive braking systems can contribute up to 21% of PM10 emissions due to traffic in urban areas. Up to 35–55% of this particulate matter remains airborne [46,47,48] while the remainder can be deposited on vehicle components or the road surface [48].

The release of heavy metals, such as Cu and Zn, typically present to control the friction coefficient and other properties of brake pad compounds of both the NAO (nonasbestos organic) or LS (low-steel) type, represents another critical issue from an environmental viewpoint that is associated with the wear of the automotive brake system [7]. In addition, the corrosive phenomena of brake discs generally made of cast iron can lead to the release of ferrous oxides or heavy metals in the composition of the friction material into the environment [49].

The introduction of BEV, HEV, and PHEV vehicles on the market has led to a marked evolution of the braking system with the introduction of electromechanical systems for energy recovery during braking. These recovery systems are generally used for braking with decelerations below 0.3 g (2.94 m/s^2^) typical of urban driving. For braking with higher decelerations and emergency braking, traditional hydraulic braking systems are used which base their operation on the use of a friction material (brake pads). It follows that the braking of vehicles in urban areas is entrusted for about 90% to the energy recovery system while only the remaining 10% involves the use of brake pads. This has a marked impact on the characteristics required for the friction materials used in BEV, HEV, and PHEV vehicles:

- reduction of the quantity of particulate matter released by the braking system (friction material);
- greater resistance to degradation due to wear compared to vehicles without a regenerative braking system related to the downsizing of the traditional braking system with less mass of the caliper and brake pad;
- reduction of corrosive phenomena of the brake disc to avoid premature wear of the disc–pad system and the phenomenon of stiction (sticking of the pad to the disc during use of the parking brake in environmental conditions of high humidity due to the corrosion of the brake disc).

## 4. Corrosion-Related Phenomena in Braking Systems

As highlighted in the first part of this review, the braking system of a vehicle is very complex since it combines different components, including the brake disk, commonly composed of gray cast iron, the brake pads containing several specific constituents, and anodized aluminum calipers [40]. This complex system can be exposed during service to extremely variable and potentially aggressive conditions, including rain, snow, Sox, and NOx-rich atmospheres and chloride-containing environments. As a result, the components of the braking systems might undergo corrosion during service. In particular, the brake discs are particularly exposed to corrosion due to the intrinsic low corrosion resistance of the gray cast iron in aggressive environments [50,51]. As discussed above, corrosion affecting the braking system of BEV, HEV, and PHEV vehicles is a critical aspect not only regarding the braking performance but also the emission of particulate matter. In particular, gray cast-iron corrosion can cause different problems ranging from an impaired aesthetic appearance to critical issues connected to the safety and reliability of the braking system. This section focuses on the main corrosion-related issues affecting the braking system of a vehicle.

### 4.1. Stick-Slip

The stick-slip in a vehicle is associated with a variation of the friction coefficient in the rusted areas of the brake discs. This can generate noise and result in a brake judder. The stick-slip phenomenon can be strongly affected by the formulation of friction material. Shin et al. [52] demonstrated that the friction film formed on the rotor surface was strongly correlated to the composition of the friction material. This affects the corrosion behavior of the disc and can trigger the formation of oxide layers with different thicknesses. Nonsteel friction materials present larger fluctuations of the friction force compared to low-steel materials during the first brake application. This force fluctuation tends to decrease for successive brake applications. The friction fluctuations were associated with the abrasive action of the corrosion products on the brake-disc surface. These corrosion products tend to remain for a longer period on the surface of the brake disk in the case of nonsteel friction materials due to their lower abrasive effect with respect to that of low-steel friction materials. This leads to a higher susceptibility to stick-slip in the case of nonsteel friction materials [52]. Park et al. [4] investigated the stick-slip behavior of nonsteel friction materials coupled with gray cast-iron discs corroded at different levels. In the case of noncorroded discs, the stick-slip amplitude was relatively small and decreased above a critical velocity for which there was a transition from stick-slip to steady sliding. Discs with corrosion products (hematite oxide particles) resulted in higher critical velocities and torque variations during stick-slip testing. This behavior was attributed to an increase in the static friction coefficient related to the transfer of oxide particles from the disc surface to the friction material. Moreover, the friction material exhibited increased wear rates during stick-slip tests with corroded discs due to a marked delamination of the friction film during high-amplitude stick-slip oscillations. The susceptibility to stick-slip can be further enhanced due to the affinity of hematite (α-Fe_2_O_3_) towards water [53]. In addition to the affinity between iron-corrosion products and water, the effect of humidity on stick-slip behavior is rather controversial. Djafri et al. [54] showed that the coefficient of friction of a cast-iron rotor sample increased with humidity up to 40% relative humidity, whereas it decreased in the interval between 40% and 90%. The decrease of the friction coefficient and wear rate of the friction material at high humidity values were attributed to the formation of a pulverulent and scarcely adherent third body of corrosion products, which could behave as solid lubricants. Similarly, Blau et al. [55] reported for corroded cast iron an initially-high frictional spike followed by a drop in friction relative to the noncorroded material. The lowered friction coefficient persisted for several drags, but it returned to friction levels similar to those of the noncorroded cast iron. The effect was explained by the formation of abrasive scales, while the decrease of the coefficient of friction was related to abrasion and ‘self-dressing’ of the surfaces by fragments of brittle corrosion products.

### 4.2. Stiction

**Figure 2:**
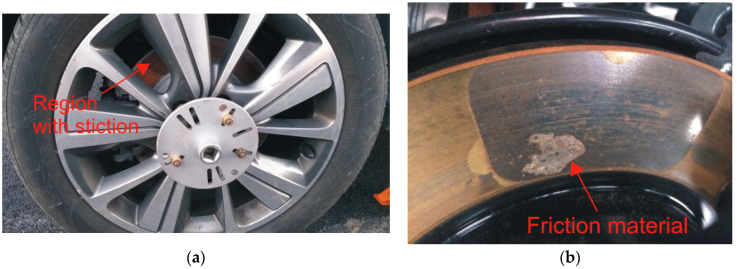
Example of corrosion stiction in an automotive braking system: (**a**) region of the brake disc showing stiction; (**b**) adhesion of friction material on the brake disc. Courtesy of ITT Italia S.r.l. (Barge (CN), Italy).

Stiction is another corrosion-related phenomenon that can affect the performance of the braking system of a vehicle [2,3,56]. It consists of the adhesion of friction material on the surface of the brake disc generating a strong bond at their interface (Figure 2). It typically occurs when the parking brake of a vehicle is activated with an applied static clamping force between the pad and the brake disc. Corrosion products generated by the corrosion of the gray cast-iron disc at the interface with the pad might penetrate into the friction material strongly increasing the possibility of generating a strong adhesion bond between the brake pad and the gray cast-iron disc [56]. This can lead to severe damage to the friction material and, in the worst cases, can prevent a vehicle from moving. The stiction phenomenon is typically observed after a prolonged period of parking, in particular in winter conditions when the pad can be exposed to chloride-containing environments that can strongly enhance the susceptibility to stiction. When stiction occurs in a vehicle, the tangential force necessary to detach the pad from the brake disc can be high enough to cause severe damage to the friction material when the parking brake is released and compromise the safety and efficiency of the braking system [3].

The stiction mechanism is generally well accepted in the literature [3,56]. The corrosion processes at the pad–disc interface lead to the formation of soluble corrosion products of the gray cast iron. The permeation of the Fe ion-containing electrolyte and the successive formation of iron oxides at the pad–disc interface after drying is responsible for the establishment of a strong bond between the pad and the rotor (stiction). The susceptibility to stiction is often evaluated by measuring the shear force necessary to detach the pad from the disc [3,56,57].

The study of the stiction phenomenon is very complicated because it is strongly affected by environmental conditions and, in particular, by the heterogeneous composition of the friction material [58]. As highlighted in the initial part of this review, friction materials contain different constituents that might potentially affect the corrosion behavior of gray cast-iron brake discs and their susceptibility to stiction. In addition, it should be considered that the formulations of friction materials are often proprietary and not disclosed by the producers. As a consequence, the investigation of the stiction phenomenon is rather scarce in the literature and a limited number of papers are available in the literature. Most of the time, research papers are focusing on the understanding the adhesion mechanism between the pad and the brake disc. The adhesion phenomena promoted by the corrosion of the gray cast iron in contact with the friction material are strongly dependent on the corrosion behavior at the pad–brake disc interface. This is extremely complex because it involves the formation of a crevice at this interface that can locally strongly modify the corrosion behavior of the gray cast iron. In addition to parameters affecting the contact between the pad and brake disc, applied pressure and surface roughness can also affect the stiction behavior. Furthermore, it is considered very important to highlight the effect of the chemical and physical properties of the friction material on stiction susceptibility.

## 5. Effect of the Chemical and Physical Properties of the Friction Material on Stiction Behaviour

**Figure 3:**
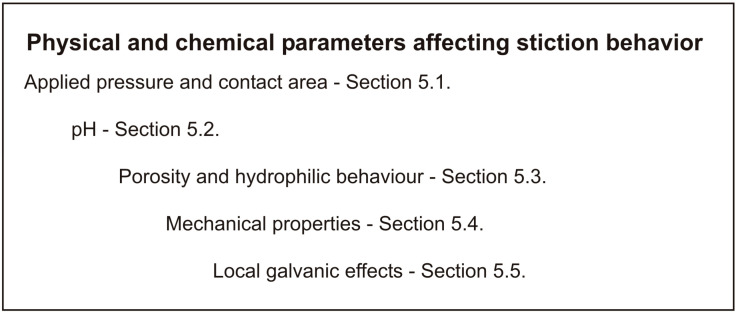
Chemical and physical properties of the friction material affecting stiction behavior.

The main physical and chemical properties of friction materials affecting corrosion-stiction behavior are given in the scheme in Figure 3.

### 5.1. Applied Pressure and Contact Area at the Rotor–Pad Interface

Gweon et al. [2,3] deeply investigated the factors affecting the stiction behavior of brake friction materials in contact with a gray cast-iron disc. In the case of low-steel and nonsteel friction materials, stiction was induced by corrosion tests carried out in an environmental chamber after soaking the friction materials in 3.5% NaCl solution [2]. The friction material and gray cast-iron specimens were assembled with a clamping device in order to control the applied pressure in the range of 1–40 bar. It was shown that the tangential force to detach the friction-material sample from the gray cast iron at the corroded interface tended to increase with the applied pressure and the time of exposure to the corrosive environment [2]. Although some variability was observed in the reported data, low-steel materials exhibited a higher susceptibility to the applied pressure during corrosion tests, while this dependency was less clear for the nonsteel materials. These results highlighted the importance of the friction materials–gray cast iron interface in stiction phenomena and it was concluded that the stiction susceptibility (stiction force) could be controlled by the contact area between the friction material and the gray cast iron at this interface, which was expected to increase at high applied pressure [2]. The contact area at this interface can be affected not only by the applied pressure but also by the surface properties of the friction material and the gray cast-iron sample. In particular, Gweon et al. [2] highlighted a clear effect of the roughness of the gray cast-iron samples employed for their stiction tests. Indeed, the stiction force increased for the polished gray cast-iron samples due to the existence of a larger contact area with the friction material at the same level of the applied pressure. In contrast, the effect of the roughness of the friction material was extremely difficult to be evaluated and no clear correlation with the stiction force could be highlighted [2]. In this case, the poor correlation was attributed to the heterogeneous structure of the friction materials, which is responsible for the random nature of the surface topography of the friction material. This aspect should be considered intrinsic to the friction material due to its complex formulation. Moreover, the roughness of the friction material can be strongly modified by braking application. The burnishing effect during brake application and the formation of a third body on the disc surface can reduce the roughness of the friction and disc materials leading to a decrease in the stiction force [2]. The effect of the contact area at the friction material–disc interface is further investigated in another work by Gweon et al. [3], which studied commercial friction materials with different roughnesses obtained by polishing starting from a preconditioned material obtained by burnishing, employing a pad-on-disc type tribometer. In this case, it was possible to prove a strong correlation between the roughness of the friction material and the stiction force. In particular, the stiction force was inversely proportional to the surface roughness of the friction material. This was associated with the increase of the contact area with the gray cast-iron rotor for friction materials with low roughness [3]. The effect of the roughness of the pad–disc interface on the stiction behavior was observed also in the work of Merlo et al. [59]. In addition, they highlighted also the importance of the third-body layer in controlling the surface roughness at the pad–disc interface.

### 5.2. pH

The pH at the friction material–disc interface can affect the corrosion behavior of the gray cast-iron disc. In particular, acid conditions can strongly increase the corrosion susceptibility of gray cast iron. Gewon et al. [2] measured the pH of distilled water containing pulverized friction material. A pH ranging between 11 and 12 was observed due to the incorporation of calcium hydroxide in the friction material. In addition, the corrosion rate of the gray cast-iron samples immersed in the solutions containing pulverized friction materials was measured by electrochemical methods. The dependency of the corrosion rate of the gray cast iron was not clear. Similarly, it was not possible to correlate the electrolyte pH to stiction forces measured for different friction materials [2,3]. It was considered that the electrolyte pH was in a narrow range due to the composition of the friction material to draw a reliable conclusion on the pH effect on stiction behavior. Similar conclusions are also reported by Merlo et al. [59]. Nevertheless, it should be considered that the electrolyte pH measured by Gewon et al. [2,3] and Merlo et al. [59] might not be representative of the conditions that are established at the pad–disc interface. Robere et al. [60] investigated the effect of the pH on the stiction behavior of two commercial NAO friction materials. The pH was modified through the addition of 3 wt% calcium hydroxide to the pad formulation. The pH of the friction formulation without calcium hydroxide was 7.1, while it was 11.9 for a 3 wt% content of calcium hydroxide in the friction material [60]. Robere et al. [60] could highlight a strong reduction of stiction susceptibility due to the pH increase determined by the addition of calcium hydroxide. This behavior was linked to the decreased corrosion susceptibility of gray cast iron in alkaline environments. Therefore, it was claimed that stiction susceptibility can be decreased considerably in friction materials with the pH adjusted to a value in the range of 11.2 to 11.9.

### 5.3. Porosity and Hydrophilic Behavior of the Friction Material

Robere et al. [60] modified the composition of commercial nonasbestos organic (NAO) friction materials in order to modify their porosity through the variation of pressure during hot stamping and the control of their resin content. In particular, it was shown that the modification of the resin content could affect the porosity of the friction material. A 3% decrease in the resin content increased the porosity from 11.7% to 17.4% while a 3% increase in the resin content led to a porosity of 9.8% [60]. The stiction test highlighted a strong correlation between the porosity of the friction materials and their propensity to stiction. In particular, it was shown that the decrease in the porosity of the friction material through an increase of the resin content strongly reduces the stiction susceptibility [60]. Passarelli et al. [56] investigated the effect of the porosity and hydrophilicity of the friction material on the stiction behavior of nonsteel-model friction materials with controlled porosity. The porosity ranged between 29% and 44%, which is significantly higher than that of commercial friction materials. This was obtained by adjusting the amount of phenolic resin and vermiculite in the formulation of the friction material. In addition, adsorption tests were carried out to qualitatively evaluate the hydrophilic behavior of the different friction materials. The testing procedure consisted of measuring the adsorption time of a 20 ml drop of deionized water positioned on the different friction materials [56]. The absorption time decreased with increasing the porosity of the friction material, clearly indicating that a high porosity promotes fast water adsorption in the friction material. Stiction tests performed with a testing procedure similar to that followed by Gweon et al. [2,3] revealed that the friction material with 29% porosity required a high shear force to be detached from the cast-iron rotor sample, while the force was significantly lower (one order of magnitude) for a friction material with 34% porosity. It was not possible to induce stiction for higher porosity levels in the friction material [56]. This behavior was explained by a low propensity to establish a thin film of electrolyte at the interface between the hydrophilic friction material and the rotor samples at high porosity levels. This hinders corrosion processes at the interface, reducing the susceptibility to stiction. Gweon et al. [3] reported a weak correlation between porosity and the stiction susceptibility of commercial friction materials. The porosity of the samples ranged between 11% and 19% in this work. Although the stiction propensity tended to decrease, increasing the porosity of the samples, the friction material with 19% porosity exhibited a high stiction force, which was correlated with the lowered cohesive strength of the pad due to its small resin content compared to other friction materials. In contrast to porosity, Gweon et al. [3] found a good correlation between the hydrophilicity of the friction material and their susceptibility to stiction. The hydrophilicity of the friction materials was investigated by contact angle measurements showing that the average stiction force tended to increase with the contact angle. In line with the work of Passarelli et al. [56], the low susceptibility to stiction of the hydrophilic pads was attributed to the limited amount of water that remained at the disc surface due to strong water absorption. The results discussed above clearly suggest that the effect of porosity and hydrophilic behavior of the friction material can affect the stiction behavior. Nevertheless, their effects are difficult to be discriminated probably due to differences in the formulations discussed in the literature and to the intrinsic complex structure of the friction materials.

### 5.4. Mechanical Properties of the Friction Material

The mechanical properties of the friction material can affect its stiction behavior. Gweon et al. [3] calculated the contact stiffness of different friction materials from load-displacement curves. It was shown that friction materials with high compressibility exhibited the highest stiction forces indicating that a low contact stiffness of the friction material promotes stiction phenomena [3]. This trend was also in good correlation with that of the shear strength of the different materials [3]. Nevertheless, corrosion phenomena at the pad–disc interface can be extremely heterogeneous. In particular, partial detachment of the friction material from the pad surface due to the penetration of iron oxides can be a localized phenomenon. This can happen when the cohesive strength of the friction material is in the same range as the shear force necessary to detach the pad from the disc. In addition, the stiffness of the friction material can strongly modify the contact area at the pad–disc interface. As considered by Gweon et al. [3], friction materials with high stiffness (low compressibility) would lead to reduced contact areas compared to less-stiff materials. Therefore, it was concluded that friction materials with higher stiffness are less prone to corrosion stiction in brake systems employing gray cast-iron discs.

### 5.5. Local Galvanic-Coupling Effects with Metal-Based Constituents

Brake pads and cast-iron discs should be carefully selected in order to find the best compromise between braking performance and corrosion resistance [61,62]. Thus, the composition of the brake pads is also a factor that may have a critical role in defining the stiction behavior at the pad–disc interface [3,62]. Indeed, the introduction of metals such as copper or zinc inside the friction material may induce galvanic coupling with the gray cast iron at the pad–disc interface affecting the stiction behavior of the material.

The presence of copper in the friction materials has often been regarded as a possible source of galvanic effects with gray cast-iron rotors due to the more noble open-circuit potential of copper relative to iron in different electrolytes [58,62,63]. Tigane et al. [58] investigated the stiction behavior of NAO friction materials containing copper in contact with gray cast iron. By means of different electrochemical methods, it was shown that the presence of copper increased the susceptibility to corrosion of the gray cast iron. This was associated with the redeposition of copper ions on the surface of the gray cast iron leading to an increase in the current density of the system [58]. This effect was combined with the peculiar configuration at the pad–disc interface that was described as a thin film of electrolyte between the friction material and the cast iron. According to their work, the copper redeposited on the cast iron can catalyze the reduction of oxygen in the thin layer of electrolyte between the disk and the pad increasing the anodic dissolution of the gray cast iron through galvanic coupling. Bandiera et al. [64] studied the galvanic coupling effects between friction materials and gray cast iron. In particular, the galvanic coupling between the metallic constituents, including copper and zinc, was investigated with an electrochemical approach. In agreement with the results reported by Tigane et al. [58], they confirmed the tendency of copper to increase the corrosion rate of gray cast-iron discs when coupled with the friction material [64]. In addition, they reported galvanic effects also between zinc and cast iron. In this case, the zinc can behave as a sacrificial anode and lead to a reduction of the corrosion rate of the gray cast iron [64].

## 6. Testing Methods to Investigate Stiction Phenomena

**Figure 4:**
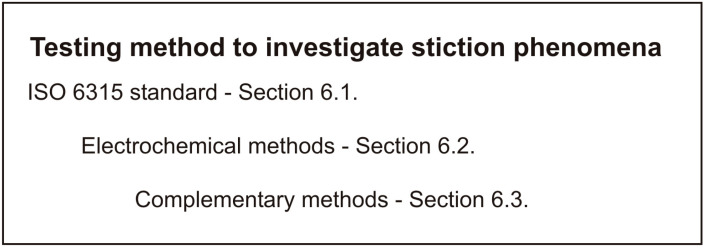
Testing methods for investigation of corrosion stiction [57].

This section considers the main testing methods reported in the literature for the investigation of corrosion stiction in automotive braking systems (Figure 4).

### 6.1. ISO 6315 Standard

**Figure 5:**
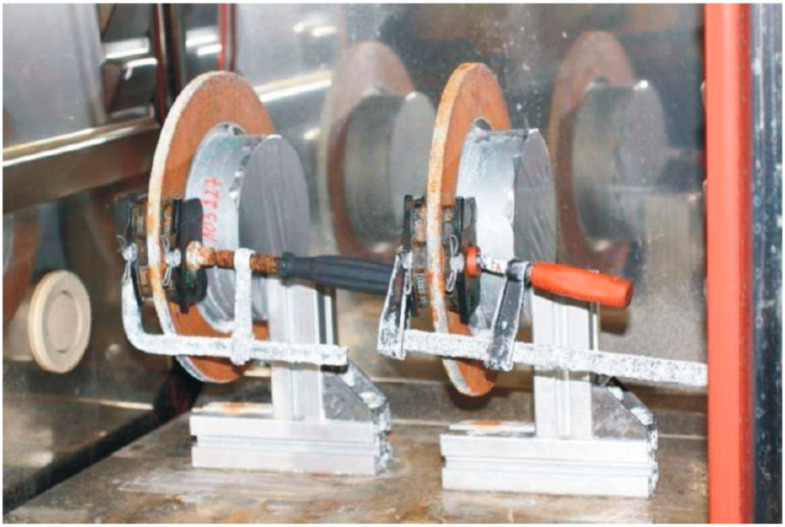
Example of exposure of a brake pad–disc couple in a humidity chamber. Courtesy of ITT Italia S.r.l. Barge (CN), Italy).

The ISO 6315 standard provides a laboratory-testing procedure to evaluate the stiction behavior of friction materials for automotive applications [57]. The stiction is induced by conditioning the friction material in contact with a disc sample in an environment promoting corrosion (Figure 5). In addition, the standard provides a method for measuring the strength of the bond between the friction material and the brake disc. The testing procedure consists of the following steps [57]:

- Exposure in a humidity chamber at 95% ± 2% relative humidity and 50 °C ± 3 °C of the friction material for 4 h and of the brake disc for 30 min (friction materials and discs must be extracted from the chamber at the same time);
- After removing surface moisture from the surfaces, the friction material must be clamped on the disc with a pressure of 2500 kPa;
- The assembled friction materials and disc must be exposed in the humidity chamber for 16 h. After these are extracted from the chamber, they should dry at room temperature for 48 h;
- After drying, the force to separate the friction material from the disc is measured in the radial direction.

A similar procedure is also reported by the standard for tests employing drums instead of discs.

**Figure 6:**
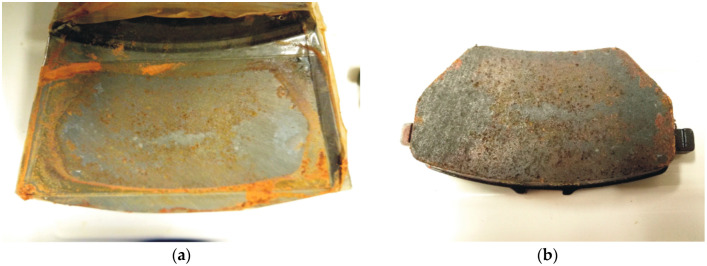
Example of corrosion stiction induced in a brake pad/disc couple following the conditioning procedure in the ISO 6315 standard: (**a**) brake disc; (**b**) brake pad. Courtesy of ITT Italia S.r.l. (Barge (CN), Italy).

Different works reported in the literature follow conditioning procedures similar to the one in the ISO 6315 standard. As an example, Robere et al. [60] soaked friction materials for 15 min in an aqueous solution containing 0.9 wt% NaCl, 0.1 wt% CaCl_2_, and 0.075 wt% NaHCO_3_. Successively, the pads are clamped on the rotors with a 5 kN clamping force and exposed for 3 days to humidity cycling between 38 °C and 100% relative humidity for 8 hours per cycle and 25 °C and 45% relative humidity for 16 hours per cycle. Similarly, Passarelli et al. [56] exposed the coupled pad–rotor in a climatic chamber at 25 °C with a relative humidity of 30% for 120 h. In general, it can be stated that methods based on conditioning in an aggressive environment followed by exposure to a humidity chamber can reproduce the stiction phenomena at a laboratory scale with a rather good reproducibility. An example is shown in Figure 6.

### 6.2. Electrochemical Methods

Since the stiction is controlled by corrosion phenomena at the pad–disc interface, several works in the literature explored the use of electrochemical methods for the investigation of the susceptibility to stiction of different friction materials [3,61,63,64,65]. In addition, a testing method based on electrochemical methods was recently patented by Sin et al. [66]. Electrochemical measurements can be carried out on single components of the braking system, including gray cast-iron rotors, aluminum calipers, and even the friction materials. Open-circuit potential (OCP) and potentiodynamic polarization (also referred to as linear-sweep voltammetry) measurements can provide information on the corrosion potential and corrosion rate of gray cast iron rotors and on the effect of alloying elements on the electrochemical behavior [51,64]. This can be done also for aluminum brake calipers [64]. Since the electrochemical investigation of metal substrates is well-established in the literature, this will not be further considered in this section. In contrast, the application of electrochemical methods to investigate friction materials is a relatively new approach. Bandiera et al. [63,64,65] performed open-circuit potential and linear-sweep voltammetry measurements on friction materials employing a typical three-electrode configuration. The friction material without an underlayer was the working electrode in the cell, graphite was employed as the counter electrode, and the reference electrode was a saturated calomel electrode [63]. Unfortunately, it is not indicated how the electrical connection of the friction material was made or if specific actions were taken to avoid wetting of the electrical connection during the electrochemical tests. The testing electrolyte was a chloride-containing aqueous solution [63]. It is specified that large areas of the working electrode (at least 20 cm^2^) are necessary to avoid artifacts related to the heterogeneous composition of the friction material. Moreover, it is mentioned that an area of the counter electrode at least twice larger than that of the working electrode is necessary to obtain reliable measurements [63]. Open circuit potential measurements on friction materials typically show that a stabilization time is necessary to obtain stationary potential values [63]. The work of Bandiera et al. [64,65] proved that OCP measurements can effectively provide information about metallic constituents in the brake pads. Linear-sweep voltammetry was also used by Bandiera et al. [63,64,65] with the same experimental setup to obtain the corrosion potential and the corrosion current density of different friction materials. In these works, a stabilization time before the initiation of the measurements is necessary in order to obtain a steady open-circuit potential before polarizing the friction material. The stabilization time was very variable according to the friction-material composition. Therefore, it was arbitrarily proposed to start the measurements when potential fluctuations were below 5 mV/h [63]. Nevertheless, it should be considered that long stabilization times in aggressive electrolytes can lead to modification of the friction materials due to redox processes. The corrosion potentials obtained by linear-sweep voltammetry in a 5 wt% NaCl solution were used by Bertasi et al. [61,64] to predict the possible galvanic effects between the friction material and gray cast iron. These works claim that a high corrosion potential of the friction material indicates a low tendency to be oxidized, while a low corrosion current density is an indication of slow oxidation kinetics. High corrosion current densities of the friction material can be ascribed to the presence of elements that can be oxidized, such as zinc or magnesium, and to its porous nature or wettability [63]. In addition, galvanic series were constructed based on the results of the linear-sweep voltammetry measurements [64]. The potential difference between the friction material and the cast iron was employed as a criterion to predict galvanic protection or galvanic corrosion of different pad–disc combinations [61]. Potential differences between the friction material and cast iron were also correlated to stiction susceptibility to show that friction materials with low corrosion current density should be employed when the friction material is the cathode in the galvanic couple with the disc, while high current densities are preferred when it is the sacrificial anode in the couple [63]. The galvanic coupling between a friction material electrically connected to cast iron was also investigated by Bandiera et al. [65] by means of a zero-resistance ammeter. The measurements were carried out with a three-electrode configuration in which the cast iron is the working electrode and the friction material is the counter electrode (with the same area for the working and counter electrodes). The reference electrode was a saturated calomel electrode. The zero-resistance ammeter was employed to measure the current flowing between the working electrode and the counter electrode. This experimental setup was used to investigate the effect of zinc in the formulation of the friction material [65]. It was shown that the presence of zinc leads to an initial regime in which the cast-iron surface is protected by the zinc that undergoes preferential oxidation. This is associated with a negative potential (about −1000 mV) of the galvanic couple. At a later stage, iron oxidation takes place when the potential of the couple becomes more positive. The transition time between these two regimes was identified as the protection time during which the Zn is able to provide galvanic protection to the cast iron [65].

Although electrochemical measurements performed on the friction materials can provide useful information for the interpretation of stiction phenomena, it should be considered that the local conditions at the pad–disc interface might be completely different from those established during free immersion in an electrolyte. Therefore, electrochemical measurements performed with a friction material in contact with the cast iron seem more representative of the investigation of stiction. This approach was recently followed by Tigane et al. [58]. The testing configuration was based on a three-electrode configuration in which the working electrode was a cast-iron sample in contact with a pad sample of cubic shape with the sides covered by an insulating paint. In this case, the electrical contact of the working electrode was on the cast iron and no contact was necessary for the friction material. Open circuit-potential measurements in 5% NaCl solution with this testing configuration exhibited a different trend compared to those carried out on the bare disk, confirming that electrochemical processes on the cast iron can be modified by contact with the friction material. In addition, the open-circuit potential of an NAO friction material containing Cu shifted the corrosion potential in the positive direction [58]. Polarization curves were also acquired in a 5% NaCl solution with and without the pad sample in contact with the cast iron. It is reported that the corrosion potential exhibits a similar shift in the positive direction to that observed in the OCP measurements when the pad is in contact with the steel. In addition, the corrosion current densities obtained by extrapolation with Tafel’s method revealed an increase in the current density of the gray cast iron when this is in contact with a pad sample. OCP and polarization curves presented by Tigane et al. [58] confirm the ability of an electrochemical approach to provide information about the effects of pad composition.

A novel approach to the investigation of stiction was also introduced in the work of Tigane et al. [58]. For the first time, they employed electrochemical impedance spectroscopy (EIS) to evaluate the behavior of a cast-iron sample in contact with a pad sample using the same setup described above for OCP and polarization measurements. The EIS spectra for the disc sample alone showed capacitive behavior with a large loop in the Nyquist plots corresponding to the charge-transfer reaction in parallel with the interfacial capacitance, which were associated with the formation of corrosion products on the cast iron surface. The spectra for the cast iron in contact with the pad sample exhibited a different shape relative to the one for the bare cast iron. The overall impedance of the system decreased, and a two time constant behavior was observed for the cast iron in contact with the pad. The capacitive loop in the high-frequency domain was associated with the charge-transfer reaction in parallel with the interfacial capacitance as for the measurements without the pad. The inductive behavior in the low-frequency domain was associated with the kinetically delayed corrosion phenomena. For longer immersion times, the shape of the EIS spectra is modified, highlighting a marked increase in the electrochemical activity of the cast iron in contact with the pad. The EIS measurements reported by Tigane et al. [58] could highlight also a marked effect of copper on the electrochemical response of the thin layer of electrolyte at the interface between the cast iron and the pad. Based on these results, a model was proposed, taking into account the dissolution of the copper contained in the pad and its redeposition on the disc, to explain the trend of the EIS spectra. Gweon et al. [3] employed the EIS technique to evaluate the size of the corroded contact area at the interface between the disc and pad in an aggressive electrolyte. It was shown that the polarization resistance at the pad–disc interface was dependent on the corroded contact area. Moreover, it was found that there is a good correlation between the polarization resistance obtained by EIS measurements and the stiction behavior of a friction material.

### 6.3. Complementary Methods

Surface characterization techniques have been employed in the literature to obtain complementary information about the phenomena leading to stiction at the pad–disc interface. Bandiera et al. [67] reviewed the main methods for the investigation of corrosion products in the case of friction materials exposed to different corrosion environments. These were representative of different conditions that can be encountered in a braking system (rain, winter driving in the presence of chlorides, and the presence of sulfuric acid due to emissions). X-ray diffraction enables a precise characterization of the oxides showing that the composition of corrosion products of gray cast iron is strongly influenced by the corrosive environment. As an example, akaganeite (β-FeOOH), magnetite (Fe_3_O_4_), and maghemite (γ-Fe_2_O_3_) are the main corrosion products in chloride-rich environments [67]. A mixture of hydroxides, including lepidocrocite (γ-FeOOH), goethite (α-FeOOH), and akaganeite (β-FeOOH), was observed after exposure to 100% relative humidity [67].

The micro-Raman spectra enable the characterization of corrosion products of cast irons showing that they consist of a complex layered structure containing different oxides, including goethite (α-FeOOH), akaganeite (β-FeOOH), and lepidocrocite (γ-FeOOH) [27]. In addition, magnetite (Fe_3_O_4_), is observed only in the inner layer of the corrosion products [27]. Bandiera et al. [67] reported that the surface of corroded cast iron in the presence of chlorides can be rather heterogeneous. Indeed, domains containing hydroxides were combined with regions containing mainly akaganeite and magnetite [67].

Since stiction is strongly related to the penetration of corrosion products of the disc in the friction material, it appears crucial to obtain information on the corrosion products at the pad–disc interface of a corroding braking system. Tigane et al. [58] combined the use of electrochemical methods with a detailed characterization of the corrosion products at the pad–disc interface by means of micro-Raman spectroscopy and energy-dispersive X-ray spectroscopy. It was reported that the corrosion products mainly consisted of iron oxides at the pad–disc interface, as expected by the corrosion of a gray cast-iron brake disc. Moreover, the composition of the iron oxides can be affected by the friction material. As an example, metallic copper deposits, as well as copper oxides, were also found at the pad/disc interface [58].

The adhesion of corrosion products at the pad–disc interface is another factor that can affect the stiction behavior of a friction material. Although this aspect is not specifically discussed in the literature, Bandiera et al. [67] highlighted that magnetite and maghemite corrosion layers show stronger adhesion to the substrate compared to other rust byproducts. The stiction mechanism that is generally accepted in the literature and the importance of the contact area at the pad–disc interface is well known in the literature [2,3,56]. A better understanding of the type of corrosion products at the pad–disc interface and their ability to establish a strong bond to the friction material is an important aspect for future research about corrosion stiction.

## 7. Approaches to Control Corrosion Stiction in Braking Systems

### 7.1. Control of Chemical-Physical Properties of the Friction Material

The discussion of the role of the chemical-physical properties on the stiction behavior of the friction material highlights that the first obvious approach to control corrosion-related issues in a braking system should consider the formulation of the friction material. The main areas of action can be summarized as follows:

- Porosity of the friction material: although the effect of porosity is rather controversial in the literature, its control through proper selection of the binder and its content is very important [2,3,56,60]. Moreover, other constituents of the friction material such as vermiculite might affect the porosity of the friction material [56];
- Mechanical properties of the friction material: friction materials with high stiffness and low compressibility are generally regarded as stiction-resistant materials due to their propensity to reduce the contact area at the pad–disc interface [2]. Proper tailoring of the mechanical properties of the friction material can significantly impact the stiction susceptibility and the extent of the damage of the friction material in case of stiction;
- pH control: the incorporation of additives, sometimes referred to as pH boosters, that can lead to an alkalinization at the pad–disc interface, can reduce the susceptibility to stiction of gray cast-iron rotors [60]. The most employed pH booster is calcium hydroxide;
- Control of local galvanic effects: it is well established that metals in the friction material can promote galvanic corrosion of the gray cast iron [58,64]. In particular, the presence of copper can be detrimental [58]. In contrast, zinc can promote the galvanic protection of the rotor and it can be considered a valid alternative to pH boosters.

Since the friction material can significantly be modified during service, the durability of the friction material plays a key role in retaining its chemical-physical properties and controlling its stiction behavior. Thus, the porosity content of the friction material and its mechanical properties should not be modified by high-temperature exposure during braking. In addition, the effect of pH boosters or metals promoting galvanic protection must be retained for long times in aggressive environments avoiding leaching phenomena or fast oxidation.

### 7.2. Surface Treatments of the Discs and Coatings

Another approach to control stiction phenomena relies on the surface treatment or the application of coatings on gray cast-iron rotors in order to improve their durability. This will also affect corrosion resistance and stiction behavior. This approach has gained increasing attention for the braking systems of electric vehicles, where the gray cast-iron rotors are more exposed to corrosion issues [68]. This is also driven by the need to reduce particulate emissions from the braking system of a vehicle [68].

In general, a reduction of the susceptibility of gray cast iron is beneficial to control corrosion stiction. This can be obtained through the surface treatment of the gray cast-iron rotors. Holly et al. [69] investigated the corrosion behavior of nitrocarburized brake rotors. It was shown that the thermochemical diffusion of nitrogen and carbon inside the cast-iron matrix leads to a marked improvement in the resistance to high-temperature oxidation, scaling, and static corrosion [69]. A similar effect can be obtained by means of a coating on the brake rotor [68]. A review of the main types of coatings for brake discs is beyond the scope of this work. Nevertheless, different types of coatings are reported in the literature, including hard chrome plating, plasma electrolytic oxidation, laser cladding, and thermal spray coatings [68]. These techniques enable the deposition of different types of coatings such as oxides (Al_2_O_3_, TiO_2_ Cr_2_O_3_, ZrO_2_, and MoO_3_), carbides (WC and Cr_3_C_2_) and cermets, Ni alloys, and cobalt–chromium alloys (stellite) [68]. As an example, Chioibasu et al. [70] reported that the corrosion rate of gray cast-iron brake discs coated with Inconel 718 by direct energy deposition was significantly lower than that of the bare discs. A similar effect is reported also for stellite coatings on gray cast-iron brake discs [71].

### 7.3. pH Boosters and Other Additives

Along with a careful selection of the brake-pads constituents, the use of specific additives is a suitable method to reduce the extent of the stiction phenomenon. The susceptibility to corrosion stiction can be effectively reduced by the incorporation of alkali materials [72]. Calcium hydroxide is the most-used anticorrosive filler belonging to this category due to its low cost [72,73]. In addition, calcium hydroxide displays low solubility, which enables limiting the fast leaching of the additive during service [72]. As discussed above, calcium hydroxide is a pH booster that leads to a local alkalinization at the pad–disc interface reducing the corrosion rate of gray cast iron. Nevertheless, the use of calcium hydroxide may also result in premature cross-linking of the polymeric binder during friction-material mixing. Encapsulation of calcium hydroxide is a possible approach to avoid premature cross-liking [73]. Precompounding elastomers with calcium hydroxide is an effective method that can be employed to isolate it from the uncured phenolic resin during friction-material molding [73]. This will also allow a controlled release of calcium hydroxide to the braking surface, even after exposure to water during service [73]. The size of the precompounding particles must be in the order of 0.5 mm to reduce their surface area, limiting the reaction of calcium hydroxide with the binder. Moreover, encapsulation with elastomers inhibits the curing catalytic effect of calcium hydroxide. Atkinson et al. [72] proposed the direct use of a reactive metal, such as calcium, lithium, sodium, or potassium, as a friction-material additive to limit corrosion phenomena in braking systems. Exposure to aqueous solutions of reactive metals leads to their reaction with water through a two-stage reaction. Initially, calcium oxide is formed by a reaction with atmospheric oxygen. This will then react with water to form calcium hydroxide only when the pad–disc interface is exposed to a corrosive environment [72]. The incorporation of the reactive metals inside the friction material may be carried out by direct addition, by means of intermetallic compounds, and/or capping in resins or oxides [72]. The unreacted metals can also affect the friction characteristics of the composite. Lamport [73] proposed also the use of metal phosphates in combination with hydroxides encapsulated in hydrophobic elastomers. In particular, it was claimed that the use of water-soluble phosphates in combination with oxides or hydroxides can be an effective way to protect the brake assembly from corrosion. Different phosphate compounds were considered, including sodium hexametaphosphate, tri-sodium phosphate, di-sodium phosphate, di-sodium polyphosphate, tri-sodium polyphosphate, or sodium pyrophosphate acid [73]. Phosphates are well known to form protective iron phosphate films on ferrous alloys. Their combined use with a hydroxide such as calcium hydroxide can lead to a synergistic effect due to the alkalinization at the pad–disc interface. It was shown that the corrosion susceptibility of a pad–disc assembly can be reduced by employing a friction material containing a 3% by volume of a water-soluble phosphate combined with a 3% by volume of calcium hydroxide [73]. Encapsulation in a hydrophobic elastomer can also contribute to reducing possible leaching effects due to contact with water or other aggressive electrolytes while preserving at the same time the thermal stability and the coefficient of friction in wetted conditions [73].

## 8. Conclusions

The main literature concerning corrosion phenomena in automotive braking systems is reviewed in this work focusing mainly on corrosion stiction and its control. This is of primary importance in the development of braking systems for application in BEV, HEV, and PHEV vehicles.

Gray cast-iron discs in automotive braking systems display an intrinsic low-corrosion resistance in aggressive environments. Corrosion-related phenomena, including stick-slip and stiction, can be exacerbated in vehicles equipped with electromechanical systems for energy recovery during braking.

- The corrosion-stiction phenomenon leads to a strong adhesion of the pad material to the brake disc under static conditions (parking brake activated) in aggressive environments. It is generally accepted that the stiction mechanism is controlled by the diffusion of corrosion products in the friction material under wet conditions and the successive formation of strong bonds with the brake disc during drying;
- The stiction behavior highlights a complex dependency on the chemical and physical properties of the friction material due to its heterogeneous chemical composition and structure. The porosity and hydrophilic behavior of brake pads play a key role in controlling the access of aggressive electrolytes at the pad–rotor interface. Local pH variations leading to the establishment of an acid environment at the pad–disk interface can strongly increase the susceptibility to corrosion of gray cast iron. Moreover, localized galvanic coupling might occur in friction materials containing copper enhancing the corrosion attack of the brake disc. Compressibility and stiffness of the friction material can significantly affect the extension of the contact area between the pad and the rotor impacting the susceptibility to corrosion stiction;
- Electrochemical techniques, including potentiodynamic polarization and electrochemical impedance spectroscopy, are useful tools for the investigation of corrosion stiction. Their combined use with surface characterization methods can lead to a better understanding of the parameters controlling this phenomenon;
- Development of friction materials with a low susceptibility to corrosion stiction should follow a complementary approach targeting an accurate selection of the constituents of the friction material, a better control of local conditions established at the pad–disc interface, and also the use of specific additives or surface treatments of gray cast-iron rotors.

## Data Availability

Not applicable.
